# Cross-Lagged Relations Between Loneliness and Cognitive Performance in Daily Life Among Older Adults

**DOI:** 10.1093/geroni/igaf122.463

**Published:** 2025-12-31

**Authors:** John Felt, Jee eun Kang, Karina Van Bogart, Martin Sliwinski, Christopher Engeland, Jennifer Grahama-Engeland

**Affiliations:** The Pennsylvania State University, University Park, Pennsylvania, United States; The Pennsylvania State University, University Park, Pennsylvania, United States; Northwestern University, Chicago, Illinois, United States; The Pennsylvania State University, University Park, Pennsylvania, United States; The Pennsylvania State University, University Park, Pennsylvania, United States; The Pennsylvania State University, University Park, Pennsylvania, United States

## Abstract

Loneliness affects 24-33% of older adults and has been associated with a 26% increased risk for developing Alzheimer’s Disease and related dementias (ADRDs). Given that ADRDs have a long preclinical period, loneliness may arise in response to earlier cognitive changes and act as a preclinical indicator. The purpose of this study was to investigate bidirectional associations between loneliness and cognitive performance in older adults. Data comes from a burst of the Einstein Aging Study, a community sample of 318 adults over the age of 70 from Bronx, NY who were dementia free at baseline. Participants completed 5 ecological momentary assessments (EMAs) per day for 14 consecutive days. Participants self-reported how lonely they felt and completed 3 brief (∼45 seconds per task) cognitive tasks at each EMA (Grid Memory, Color Shapes, and Symbol Search). We used dynamic structural equation modeling (DSEM) to test the cross-lagged relations between loneliness and cognitive performance on the three tasks (∼every 3-4 hours). Results indicated that higher cognitive performance at one EMA was associated with lower levels of loneliness on the following EMA for the proportion of shapes correctly identified in the Color Shape task. There was also some evidence that higher levels of loneliness were associated with slower reaction time on the Symbol Search task. Further, higher levels of loneliness at one EMA were associated with slower performance on the Color Shape task. These results will be discussed in the context of pre-clinical mechanisms and additional results on multiple burst data will be presented.

